# Exosomes Secreted From Bone Marrow Mesenchymal Stem Cells Attenuate Oxygen-Glucose Deprivation/Reoxygenation-Induced Pyroptosis in PC12 Cells by Promoting AMPK-Dependent Autophagic Flux

**DOI:** 10.3389/fncel.2020.00182

**Published:** 2020-07-17

**Authors:** Qing Zeng, Yuqing Zhou, Donghui Liang, He He, Xiaoli Liu, Rui Zhu, Meimei Zhang, Xun Luo, Yao Wang, Guozhi Huang

**Affiliations:** ^1^Department of Rehabilitation Medicine, Zhujiang Hospital, Southern Medical University, Guangzhou, China; ^2^Rehabilitation Medical School, Southern Medical University, Guangzhou, China; ^3^Department of Traditional Chinese Medicine, Zhujiang Hospital, Southern Medical University, Guangzhou, China; ^4^Kerry Rehabilitation Medicine Research Institute, Shenzhen, China; ^5^Shenzhen Sanming Project Group, Spaulding Rehabilitation Hospital, Harvard Medical School, Charlestown, MA, United States; ^6^Department of Rehabilitation Medicine, Shenzhen Dapeng New District Nan’ao People’s Hospital, Shenzhen, China

**Keywords:** autophagy, exosomes secreted from bone marrow mesenchymal stem cells, cerebral ischemia/reperfusion, pyroptosis, nucleotide-binding domain leucine-rich repeats family protein 3

## Abstract

**Background**: Cerebral ischemia–reperfusion (I/R) injury can lead to severe dysfunction, and its treatment is difficult. It is reported that nucleotide-binding domain and leucine-rich repeat family protein 3 (NLRP3) inflammasome-mediated cell pyroptosis is an important part of cerebral I/R injury and the activation of autophagy can inhibit pyroptosis in some tissue injury. Our previous study found that the protective effects of bone marrow mesenchymal stem cells (BMSCs) in cerebral I/R injury may be associated with the regulation of autophagy. Recent studies have demonstrated that exosomes secreted from BMSCs (BMSC-Exos) may play an essential role in the effective biological performance of BMSCs and the protective mechanism of BMSC-Exos is associated with the activation of autophagy and the remission of inflammation, but it has not been reported in studies of cerebral I/R injury. We aimed to investigate the effects of BMSC-Exos on cerebral I/R injury and determine if the mechanism is associated with the regulation of pyroptosis and autophagic flux.

**Method**: PC12 cells were subjected to oxygen-glucose deprivation/reoxygenation (OGD/R) to induce cerebral I/R *in vitro* and were cocultured with BMSC-Exos. Cell viability was determined with CCK-8 and lactate dehydrogenase (LDH) detection kits. Scanning electron microscopy (SEM), Hoechst 33342/propidium iodide (PI) double staining, 2′,7′-dichlorodihydrofluorescein diacetate assay, immunofluorescence, Western blot, and Enzyme-linked immunosorbent assay (ELISA) were used to detect cell pyroptosis. Furthermore, transmission electron microscopy (TEM), GFP-RFP-LC3 adenovirus transfection, and Western blot were used to detect autophagic flux and its influence on pyroptosis. Finally, coimmunoprecipitation was used to detect the binding interaction between NLRP3 and LC3.

**Results**: BMSC-Exos increased cell viability in OGD/R. The inhibitory effect of BMSC-Exos on pyroptosis was comparable to the NLRP3 inhibitor MCC950 and was reversed by NLRP3 overexpression. Furthermore, BMSC-Exos promoted autophagic flux through the AMP-activated kinase (AMPK)/mammalian target of the rapamycin pathway, whereas chloroquine, AMPK silencing, and compound C blocked the inhibitory effect on pyroptosis.

**Conclusions**: BMSC-Exos can protect PC12 cells against OGD/R injury *via* attenuation of NLRP3 inflammasome-mediated pyroptosis by promoting AMPK-dependent autophagic flux.

## Introduction

Ischemic stroke accounts for the majority of stroke cases and is the second leading cause of death worldwide. Ischemic stroke is a common cerebrovascular disease with high morbidity, mortality, and disability (Stonesifer et al., 2017; Wang et al., 2017; Campbell et al., 2019). In the clinical setting, thrombolytic therapy to restore the blood supply is a common treatment for ischemic stroke. However, blood reperfusion after cerebral ischemia often causes oxidative stress, inflammation, and other adverse effects, which can aggravate the cerebral injury and lead to further dysfunction (Jayaraj et al., 2019; Lambertsen et al., 2019). Therefore, it is necessary to explore more effective treatments to reduce the cerebral ischemia/reperfusion (I/R) injury and promote the repair of nerve function. Cerebral I/R injury involves complex pathophysiological processes, including autophagy (Zhang D. M. et al., 2019), apoptosis (Chen et al., 2017), oxidative stress (Rana and Singh, 2018), pyroptosis (Zhu et al., 2019), ion homeostasis imbalance (Gu et al., 2019), and acidosis (Fan et al., 2014). Among them, pyroptosis has recently been discovered as a pro-inflammatory programmed cell death process that plays an important role in cerebral I/R injury (Xia et al., 2018; Zhu et al., 2019). Recent studies have suggested that activation of autophagy has a potential therapeutic effect on cerebral I/R injury (Yao et al., 2019), and the mechanism of autophagy may be related to the inhibition of pyroptosis in some tissue injury (Li et al., 2019), which still remains unclear in cerebral I/R injury.

Bone marrow mesenchymal stem cell (BMSC) transplantation has been shown to promote the recovery of nerve function after cerebral ischemia (Stonesifer et al., 2017; Zhang Q. et al., 2019). Our previous study found that the protective effects of BMSCs in cerebral I/R injury may be associated with the regulation of autophagy *via* the PI3K/Akt/mTOR signaling pathway (He et al., 2019). Recent studies have demonstrated that exosomes secreted from BMSCs (BMSC-Exos) may play important roles in the effective biological performance of BMSCs (McBride et al., 2017; Lazar et al., 2018). Moreover, BMSC-Exos affect the biological characteristics of target cells through their interaction with specific ligand receptors, the transfer of receptors between cells, and the transfer of proteins and RNAs to target cells (Hou et al., 2020). Moreover, without any cytological characteristics such as proliferation and differentiation of BMSCs, BMSC-Exos have relatively stable biological characteristics, which reduce the risk of BMSC transplantation and make it possible to replace BMSCs for more effective treatment of cerebral I/R injury. In addition, BMSC-Exos have been shown to protect against myocardial I/R injury and inhibit myocardial infarction pathogenesis by regulating autophagy (Zou et al., 2019). Therefore, we hypothesized that BMSC-Exos may have a similar effect in cerebral I/R injury and the mechanism may be related to autophagy and pyroptosis.

In the present study, PC12 cells were subjected to OGD/R to stimulate cerebral I/R injury *in vitro* to investigate the effect of BMSC-Exos in cerebral I/R injury as well as the role of the AMP-activated kinase (AMPK)-dependent autophagic flux in the protection of BMSC-Exos against nucleotide-binding domain and leucine-rich repeat family protein 3 (NLRP3) inflammasome-mediated pyroptosis.

## Materials and Methods

### Cell Culture

Rat pheochromocytoma (PC12) cells were obtained from Jennio Biotech (Guangzhou, China) and were maintained in RPMI-1640 (Gibco, Gaithersburg, MD, USA) medium supplemented with 10% fetal bovine serum (FBS), 100 U/ml penicillin, and 100 mg/ml streptomycin in a 37°C incubator with 5% CO_2_. When the cell density reached approximately 90%, the cells were detached with 0.02% EDTA/0.25% trypsin. Cells in the logarithmic phase and those that demonstrated good growth were used for subsequent experiments.

### Oxygen-Glucose Deprivation/Reoxygenation (OGD/R) for *in vitro* Cerebral I/R

OGD/R has been recognized as an *in vitro* model for simulating I/R injury (Chen et al., 2020). PC12 cells have been widely used in neurophysiological and pathological research (Koubi et al., 2005). To mimic cerebral I/R *in vitro*, PC12 cells were subjected to OGD/R according to prior demonstrations (Ren et al., 2019). Briefly, cells were washed three times with phosphate-buffered saline (PBS; Gibco, Gaithersburg, MD, USA) and incubated for 12 h in glucose-free Dulbecco’s modified Eagle’s medium (DMEM; Gibco, Gaithersburg, MD, USA) medium under hypoxic conditions (1% O_2_, 94% N_2_, and 5% CO_2_). The cells were then incubated under normoxic conditions (95% air and 5% CO_2_) in RPMI-1640 medium for 1 h for reoxygenation. The cells were cocultured with BMSC-Exos (10 μg/ml), chloroquine (Cq; Sigma, Georgetown, SC, USA; 5 μM) or compound C (Sigma, Georgetown, SC, USA; 5 μM) during OGD/R.

### BMSC Isolation and Characterization

Primary BMSCs were isolated from male rats (80–100 g) as described previously (He et al., 2019). Cell pellets were cultured in DMEM/F-12 (1:1; Gibco, Gaithersburg, MD, USA) supplemented with 10% FBS, 100 U/ml penicillin, and 100 mg/ml streptomycin in a 37°C incubator with 5% CO_2_. When the cells reached 90% confluence, the BMSCs were detached with 0.02% EDTA/0.25% trypsin. For phenotypic analysis, the expression of CD29, CD90, CD44, and CD45 were evaluated. An immunoglobulin G (IgG)-matched isotype was used as the internal control for each antibody. BMSCs at passages 3 (P3)–P8 were used for subsequent experiments.

### BMSC-Exos Isolation and Characterization

BMSC-Exos were isolated from the cell culture supernatants of BMSCs. Before collecting the culture medium, the BMSCs were washed twice with PBS and the medium was changed to serum-free medium. The medium was then collected and centrifuged at 2,000× *g* for 30 min, 10,000× *g* for 30 min, and 100,000× *g* for 4 h at 4°C using an ultracentrifuge. The isolated exosomes were washed once with PBS and resuspended for further characterization by transmission electron microscopy (TEM), Western blot, and NanoSight NTA technology according to international standards (Théry et al., 2018).

### Cell Viability Assays by CCK-8

Cell viability was detected using the CCK-8 kit, according to the manufacturer’s instructions (TransGen Biotech, China). Briefly, the culture medium was removed and CCK-8 (10%, 100 μl/well) was added to each well and incubated for 1 h. A microplate reader was used to measure the absorbance OD value at 450 nm.

### Evaluation of Lactate Dehydrogenase (LDH) Release

LDH released into the cell culture supernatants was determined with the LDH detection kit, according to the manufacturer’s instructions (KeyGENBioTECH, China). A microplate reader was used to measure the absorbance OD value at 440 nm.

### Assessment of Reactive Oxygen Species (ROS) Levels

A 2′,7′-dichlorodihydrofluorescein diacetate (DCFH-DA) kit (Sigma, Georgetown, SC, USA) was used to detect ROS levels. After exposure to OGD/R, the cells were washed with PBS and treated with DCFH-DA (20 μM) for 30 min at 37°C in the darkness. Images were collected using a confocal microscope (Leica Microsystems, Wetzlar, Germany).

### Scanning Electron Microscopy (SEM)

For SEM, cells were seeded on glass slides. After treatment, three glass slides were selected for each group and the 2.5% glutaraldehyde solution was added to the slides for fixation. For inspection, the specimens were commissioned to the Central Laboratory of Southern Medical University for post-processing.

### Hoechst 33342/Propidium Iodide (PI) Double Staining

The Hoechst 33342 stain can penetrate the complete cell membranes, whereas the PI stain cannot penetrate normal or apoptotic cells with intact cell membranes but can enter cells through pyroptosis-related pores in the membranes. PC12 cells in each group were stained with Hoechst 33342 (Sigma, Georgetown, SC, USA; 10 μg/ml), followed by PI (Sigma, Georgetown, SC, USA; 1 μg/ml) for 15 min at 37°C. An inversion fluorescence microscope (Leica Microsystems, Wetzlar, Germany) was used to collect the images and the percentage of PI-positive cells was calculated.

### Enzyme-Linked Immunosorbent Assay (ELISA)

The levels of interleukin-1β (IL-1β) in the cell culture supernatants were detected by ELISA kits, according to the manufacturer’s instructions (Cloud-Clone Crop, China).

### Immunofluorescence Staining

After being treated with 4% buffered paraformaldehyde for 15 min, the cells were blocked with 0.2% Triton-X and 1% BSA for 1 h. The cells were then incubated overnight with the primary antibody against the N-terminal of gasdermin D (GSDMD-N; CST, USA) at 4°C. The cells were subsequently incubated for 2 h with the appropriate secondary antibody at room temperature. DAPI (Sigma, Georgetown, SC, USA) was used to stain the nuclei, and images were captured using a fluorescence microscope (Leica Microsystems, Wetzlar, Germany).

### TEM

TEM was used to observe the autophagic flux of the cells. The medium was removed, and the cells were scraped off, followed by centrifugation at 2,000 rpm for 5 min. The cells were then fixed in 2.5% glutaraldehyde for 2 h at room temperature and kept at 4°C until further analysis. The specimens were subsequently commissioned to the Central Laboratory of Southern Medical University for post-processing.

### Transfection of GFP-RFP-LC3 Adenoviruses

Cells were cultured in confocal dishes with 8 × 10^4^ cells per well and then transiently transfected with GFP-RFP-LC3 adenoviruses, according to the manufacturer’s instructions (GeneChem, China). After treatment, the cells were washed with PBS, fixed with 4% buffered paraformaldehyde, stained with Hoechst 33342, and observed under a confocal microscope (Leica Microsystems, Wetzlar, Germany). The numbers of yellow puncta (GFP+RFP+) and red puncta (GFP-RFP+) were counted for each cell, representing the autophagosomes and autolysosomes, respectively.

### Protein Extraction and Western Blot Analysis

After being washed with cold PBS, the cells were lysed with RIPA buffer containing protease-inhibitor cocktail and PMSF. SDS-PAGE was then used to separate equal amounts of protein, and the gels were transferred onto PVDF membranes. The membranes were then blocked with 5% nonfat milk for 1 h at room temperature, followed by an overnight incubation with the primary antibody solution at 4°C. After three washes with TBST, the membranes were incubated with the horseradish peroxidase-linked secondary antibodies for 2 h at room temperature. Immunoreactive bands were visualized by enhanced chemiluminescence and quantified by ImageJ software. β-Actin was used as the internal standard. The antibodies used in this study included anti-NLRP3 (1:1,000, Abcam, Eugene, OR, USA), anti-caspase-1 (1:1,000, Proteintech, China), anti-GSDMD (1:1,000, CST, USA), anti-AMPK (1:1,000, Bioss, China), anti-p-AMPK (1:1,000, CST, USA), anti-mTOR (1:1,000, Bioss, China), anti-p-mTOR (1:1,000, Abcam, Eugene, OR, USA), anti-LC3B (1:1,000, CST, USA), and anti-P62 (1:1,000, CST, USA).

### Construction of Plasmids and Transfection

To overexpress NLRP3, pGV102-NLRP3 plasmids were constructed by GeneChem (Shanghai, China) and transfected into PC12 cells. The pCMV-MCS-SV40-Neomycin plasmids acted as the control group. Transient transfection was performed by the Lipofectamine 3000 reagent (Invitrogen, USA) according to the manufacturer’s instructions.

### Real-Time Quantitative Polymerase Chain Reaction (RT-qPCR)

The effect of pNLRP3 transfection was determined by RT-qPCR. Total RNA was extracted using the TRIzol regent kit (TaKaRa Bio, Japan), and cDNA was prepared by reverse transcription. PCR was performed on a 7500 FAST Real-Time PCR System (Applied Biosystems, Foster City, CA, USA) with specific primers and the RT-qPCR Assay Kit (TaKaRa Bio, Japan). Primer sequences of the targeted genes used in this study were as follows: NLRP3 (5′-ATTACCCACCCGAGAAAGG-3′, forward; 5′-CATGAGTGTGGCTAGATCCAAG-3′, reverse) and β-actin (5′-CACCCGCGAGTACAACCTTC-3′, forward; 5′-CCCATACCCACCATCACACC-3′, reverse). NLRP3 expression levels were normalized for expression of β-actin and expressed as the fold ratio compared with the control group.

### Short Interfering RNA (siRNA) Transfection

PC12 cells were transfected with siRNA directed against AMPK, with scrambled siRNA used as control. Both specific and control siRNAs were obtained from Obio Technology (Shanghai, China). After siRNA transfection for 48 h, the cells were subjected to OGD/R and then collected for subsequent analyses. Transient transfection was performed using Lipofectamine 3000 reagent (Invitrogen, USA) according to the manufacturer’s instructions.

### Coimmunoprecipitation (Co-IP) Assays

For Co-IP assays, the PC12 cells were lysed in RIPA buffer containing protease inhibitors. Approximately 100 μg of total protein was mixed with a suitable amount (2 μg) of the anti-LC3B antibody (Abcam, Eugene, OR, USA) or the anti-normal IgG antibody. Protein A+G agarose was added to the lysate mixture, and the solution was incubated with gentle rocking at 4°C overnight. The samples were then pelleted by centrifugation, retrieved and suspended in 50 μl of SDS-PAGE buffer for immunoblotting. The obtained samples were then subjected to Western blot with the anti-LC3B (Abcam, Eugene, OR, USA) and anti-NLRP3 antibodies (Abcam, Eugene, OR, USA).

### Statistical Analyses

All experiments in the “Materials and Methods” section were conducted in triplicate. GraphPad Prism 5.0 was used for statistical analysis. A *p*-value < 0.05 was considered statistically significant. Homogeneity testing and one-way analysis of variance were used to evaluate the differences among the groups. The data are expressed as the mean ± SEM.

## Results

### Characterization of BMSCs and BMSC-Exos

**Figure 1:**
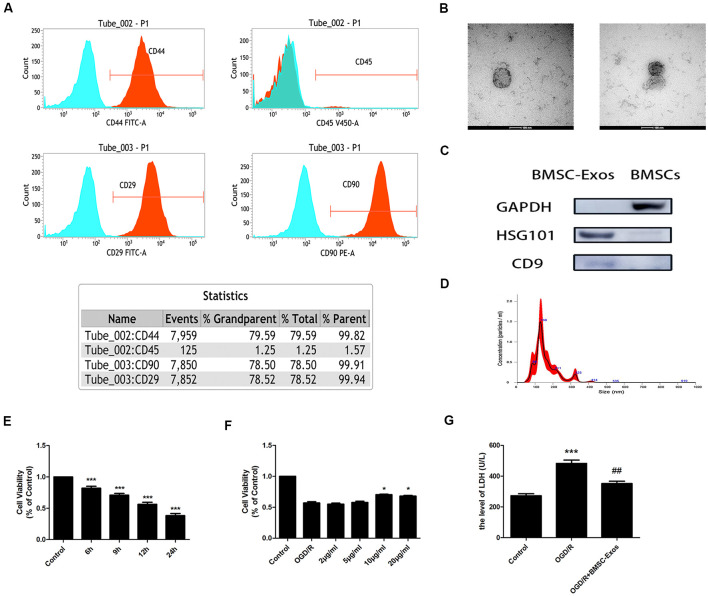
Characterization of bone marrow mesenchymal stem cells (BMSCs) and exosomes from BMSCs (BMSC-Exos); BMSC-Exos increase cell viability following oxygen-glucose deprivation/reoxygenation (OGD/R). **(A)** Flow cytometry analyses indicated that BMSCs were positive for CD29, CD44, and CD90 but were negative for CD45. **(B)** Round morphologies of BMSC-Exos were demonstrated by tandem electron microscopy (TEM); scale bar = 100 nm. **(C)** Western blot analyses showed that the BMSC-Exos were positive for the specific exosome surface markers TSG101 and CD9. **(D)** NanoSight NTA analysis indicated that the diameters of BMSC-Exos were around 100 nm. **(E)** A significant reduction in cell viability was observed in OGD 6-h, 9-h, 12-h, and 24-h groups (*n* = 3). **(F)** BMSC-Exos enhanced cell viability in a dose-dependent manner (*n* = 3). **(G)** BMSC-Exos reduced lactate dehydrogenase (LDH) release following OGD/R (*n* = 3). *p < 0.05, ***p < 0.001 vs. control group; ^##^*p* < 0.01 vs. OGD/R group.

Our results indicated that the expansion potential of P4 was positive for CD29, CD44, and CD90 but negative for CD45 (Figure 1A). The purity of the BMSCs in the culture was determined to be up to 99%, confirming that they met the standard for defining BMSCs and their use in further experiments.

TEM, Western blot, and NanoSight NTA were performed to comprehensively characterize the purified nanoparticles derived from BMSCs. As shown in Figure 1B, TEM demonstrated that the BMSC-Exos exhibited a round-shaped morphology, indicating the presence of exosomes. In addition, Western blot showed that the BMSC-Exos were positive for the specific exosome surface markers TSG101 and CD9 (Figure 1C). NanoSight NTA indicated that the diameter of the BMSC-Exos was around 100 nm (Figure 1D). Collectively, these analyses confirmed that the BMSC-Exos were successfully isolated and identified.

### BMSC-Exos Increase Cell Viability in OGD/R

CCK-8 assay results showed that the cell viability of the OGD/R group decreased significantly in a time-dependent manner. Compared with the control group, the cell viability was reduced significantly in the OGD 6-h, 9-h, 12-h, and 24-h groups (Figure 1E). The cell viability of the OGD 12-h group decreased to about 50%; thus, this group was selected as the OGD/R group. These results indicated that treatment with BMSC-Exos effectively enhanced the viability of PC12 cells in a dose-dependent manner (Figure 1F). There was no significant difference between BMSC-Exos at 10 and 20 μg/ml; thus, the former was selected as the intervention dose. In addition, the LDH value was shown to increase after OGD/R treatment. Furthermore, the LDH release of the cell supernatant was reduced after BMSC-Exos treatment when compared with the OGD/R group (Figure 1G). These results showed that BMSC-Exos could significantly improve the viability of PC12 cells after OGD/R, suggesting that BMSC-Exos had a protective effect against OGD/R injury in PC12 cells.

### BMSC-Exos Reduce the NLRP3 Inflammasome-Mediated Pyroptosis Induced by OGD/R

#### Morphological Changes Observed by SEM

**Figure 2:**
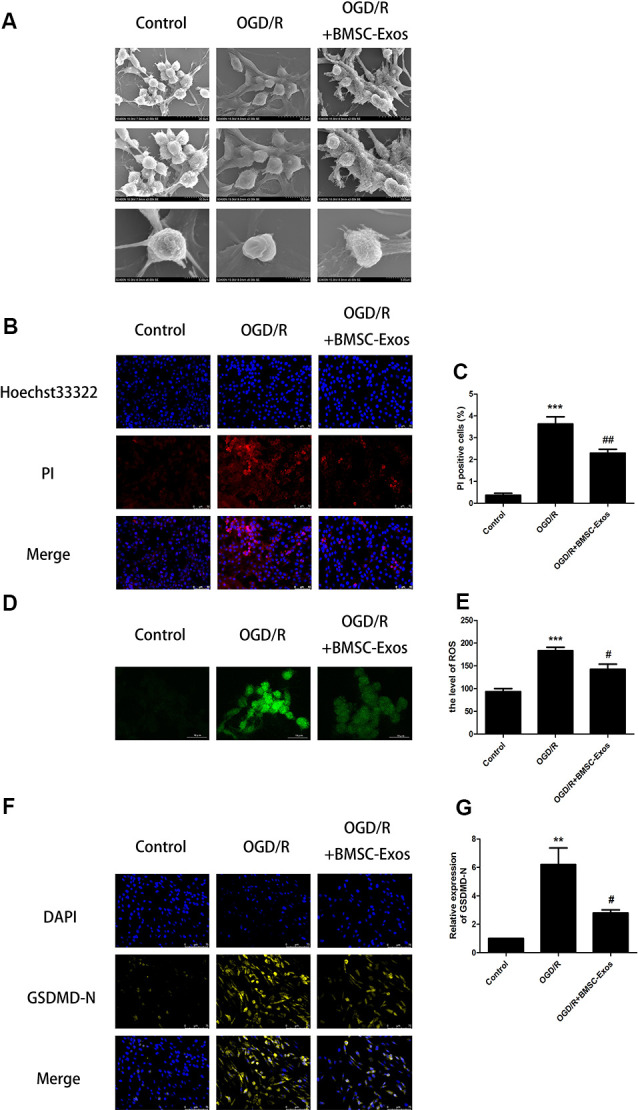
BMSC-Exos reduce pyroptosis induced by OGD/R. **(A)** Morphological changes of PC12 cells were observed using scanning electron microscopy (SEM); scale bar = 20/10/5 μm. **(B)** Representative images of Hoechst 33342/propidium iodide (PI) staining; scale bar = 50 μm. **(C)** PI-stained cells (red puncta) were quantified as the percentage of red puncta/total puncta signals in merged images (*n* = 3). PI-stained areas were lower in the BMSC-Exos group than in the OGD/R group (*n* = 3). **(D)** Representative images of reactive oxygen species (ROS); scale bar = 10 μm. **(E)** ROS levels are presented relative to control levels (*n* = 3). ROS levels in the BMSC-Exos group were lower than in the OGD/R group. **(F)** Representative immunofluorescence images; scale bar = 75 μm. **(G)** Relative expression levels of N-terminal of gasdermin D (GSDMD-N) were lower in the BMSC-Exos group than in the OGD/R group (*n* = 3). **p < 0.01, ***p < 0.001 vs. control group, ^#^*p* < 0.05; ^##^*p* < 0.01 vs. OGD/R group.

As shown in Figure 2A, SEM showed that the PC12 cells of the control group had clear outlines, long protuberances, and tight cell connections. However, in the OGD/R group, the cell membranes had lost their integrity. In addition, the cells had swollen and became flat and the surface villi were significantly reduced, which is consistent with the previously reported characteristics of late pyroptosis (Herr et al., 2020). In the BMSC-Exos treatment group, the cell contours became clearer; the surface villi increased and became denser, with longer protuberances, indicating that the characteristics of pyroptosis were significantly reduced.

#### Hoechst 33342/PI Double Staining

In the process of pyroptosis, pores can be formed in the cell membranes and result in the release of cellular contents and positive staining of dead cells, which can be determined by Hoechst 33342/PI double staining (Wu et al., 2018). Our results indicated that the PI staining proportion was increased significantly in the OGD/R group when compared with the control group. In addition, the PI staining proportion was decreased significantly in the BMSC-Exos group when compared with the OGD/R group, indicating that pyroptosis was significantly inhibited (Figures 2B,C).

#### ROS Levels

ROS is essential for NLRP3 inflammasome activation and ROS activation-induced NLRP3 inflammasome triggering caspase-1-dependent pyroptosis plays an important role in I/R injury (Liao et al., 2019). Our results showed that ROS levels were significantly increased in the OGD/R group. In addition, ROS levels were significantly decreased in the BMSC-Exos group compared with the OGD/R group, suggesting that BMSC-Exos significantly inhibited the activation of NLRP3 (Figures 2D,E).

#### Activities of NLRP3, Cleaved Caspase-1, GSDMD-N, and IL-1β

**Figure 3:**
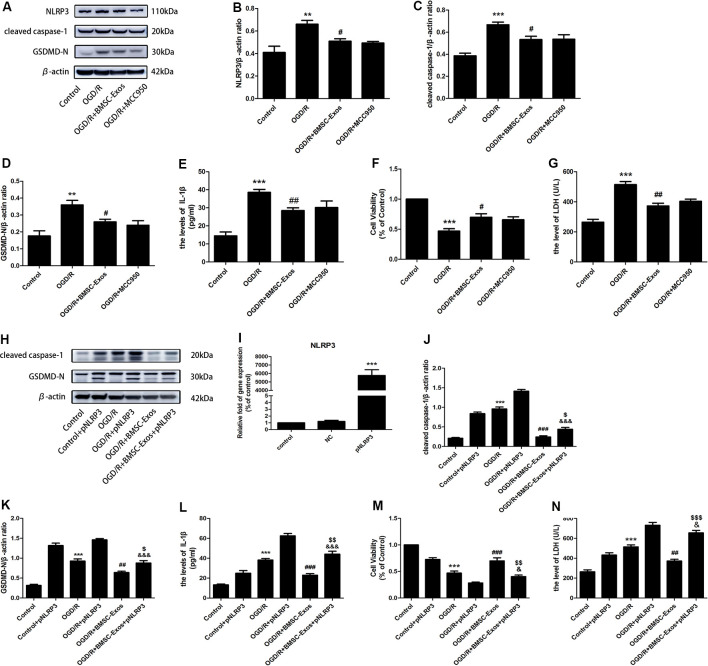
BMSC-Exos reduce nucleotide-binding domain and leucine-rich repeat family protein 3 (NLRP3) inflammasome-mediated pyroptosis following OGD/R. **(A)** Representative Western blots of NLRP3, cleaved caspase-1, and GSDMD-N. **(B–D)** Expression levels of NLRP3, cleaved caspase-1, and GSDMD-N in the BMSC-Exos group were higher than in the OGD/R group, whereas no significant difference was observed between BMSC-Exos and MCC950 groups (*n* = 3). **(E)** Interleukin-1β (IL-1β) levels in the BMSC-Exos group were higher than in the OGD/R group, whereas no significant difference was observed between BMSC-Exos and MCC950 groups (*n* = 3). **(F)** No significant difference in cell viability was observed between BMSC-Exos and MCC950 groups (*n* = 3). **(G)** No significant difference in LDH release was observed between BMSC-Exos and MCC950 groups (*n* = 3). **(H)** Representative Western blots of cleaved caspase-1 and GSDMD-N. **(I)** Real-time quantitative polymerase chain reaction (RT-qPCR) showing the effects of pNLRP3 transfection (*n* = 3). **(J,K)** Expression levels of cleaved caspase-1 and GSDMD-N were higher in the BMSC-Exos+pNLRP3 group than in the BMSC-Exos group (*n* = 3). **(L)** IL-1β levels were higher in the BMSC-Exos+pNLRP3 group than in the BMSC-Exos group (*n* = 3). **(M)** Cell viability in the BMSC-Exos+pNLRP3 group was lower than in the BMSC-Exos group (*n* = 3). **(N)** LDH release in the BMSC-Exos+pNLRP3 group was higher than in the BMSC-Exos group (*n* = 3). **p < 0.01, ***p < 0.001 vs. control group; ^#^*p* < 0.05, ^##^*p* < 0.01, ^###^*p* < 0.001 vs. OGD/R group; ^$^*p* < 0.05, ^$$^*p* < 0.01, ^$$$^ *p* < 0.001 vs. OGD/R+BMSC-Exos group; ^&^*p* < 0.05, ^&&&^*p* < 0.001 vs. OGD/R+pNLRP3 group.

Western blot results showed that the expression levels of NLRP3, cleaved caspase-1, and IL-1β were increased significantly in the OGD/R group and these increased levels were alleviated with BMSC-Exos treatment. We also used immunofluorescence and Western blot to detect the expression levels of GSDMD-N, a key enzyme in the process of pyroptosis. Our results suggested that BMSC-Exos inhibited the expression of GSDMD-N when compared with the OGD/R group (Figures 2F,G, 3A,G).

To identify whether NLRP3 inflammasome-mediated pyroptosis was involved in the neuroprotection of BMSC-Exos, the PC12 cells were treated with and without the NLRP3 inhibitor MCC950 under OGD/R conditions. Our results showed that suppressing the activation of NLRP3 with BMSC-Exos was similar to the effect of the inhibitor MCC950 (Figures 3A–G). However, NLRP3 overexpression gave rise to a significant increase in pyroptosis-related proteins, but the levels of cleaved caspase-1, GSDMD-N, and IL-1β were decreased in the OGD/R+BMSC-Exos+pNLRP3 group when compared with the OGD/R+pNLRP3 group (Figures 3H–N). These results suggested that BMSC-Exos play protective roles in the NLRP3 inflammasome-mediated pyroptosis induced by OGD/R.

### BMSC-Exos Promote Autophagic Flux in OGD/R Through the AMPK/mTOR Pathway

**Figure 4:**
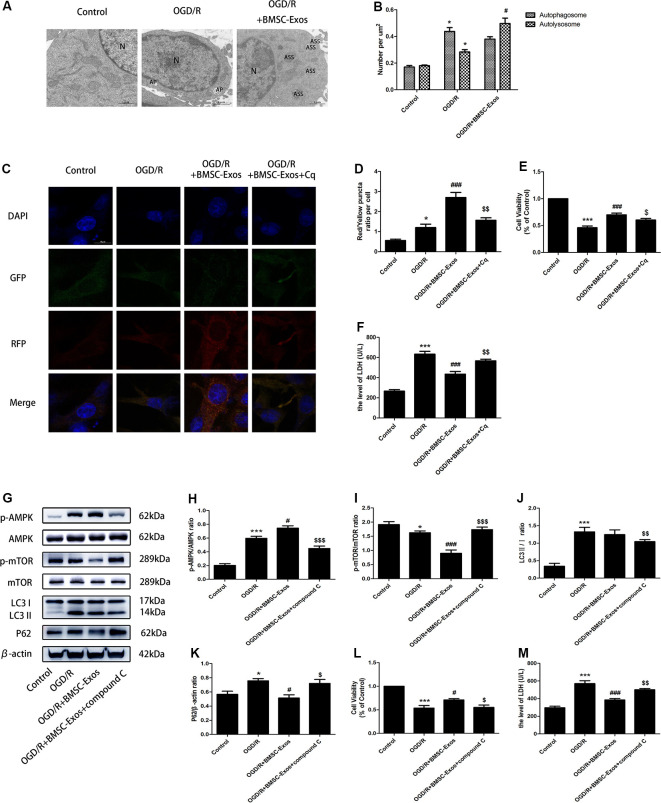
BMSC-Exos promote autophagic flux through the AMP-activated kinase (AMPK)/mammalian target of rapamycin (mTOR) pathway following OGD/R. **(A)** Autophagic flux was detected using TEM. Typical cytoplasms and nuclei (N) in the control group; double-membrane autophagosomes (AP) were observed in the OGD/R group. Autolysosomes were darkly stained, indicating that autolysosomes (ASS) are activated in the OGD/R+BMSC-Exos group; scale bar = 1 μm. **(B)** Quantitative analysis of numbers of autophagosomes and autolysosomes in each treatment group (*n* = 3). Autophagosomes and autolysosomes were more numerous in the OGD/R group than in the control group, whereas larger numbers of autolysosomes were detected in the BMSC-Exos group. **(C)** Representative images of GFP-RFP-LC3 staining; scale bar = 10 μm. **(D)** Autophagy was quantified as the ratio of red puncta (GFP-RFP+) to yellow puncta (GFP+RFP+) in each cell. This ratio was higher in the BMSC-Exos group than in control and OGD/R groups (*n* = 3). **(E,F)** The autophagy inhibitor Cq reversed cell viability and LDH release (*n* = 3). **(G)** Representative Western blots of p-AMPK, AMPK, p-mTOR, mTOR, LC3 II/I and P62. **(H–K)** LC3 II/I and p-AMPK/AMPK expression increased, although P62 and p-mTOR/mTOR expression levels decreased in the OGD/R group when compared with the control group (*n* = 3). LC3 II/I expression did not change significantly after BMSC-Exos treatment, whereas p-AMPK/AMPK expression increased further and p-mTOR/mTOR and P62 expression levels decreased (*n* = 3). **(L,M)** The AMPK inhibitor compound C reversed the effects of OGD/R on cell viability and LDH release (*n* = 3). *p < 0.05, ***p < 0.001 vs. control group; ^#^*p* < 0.05, ^###^*p* < 0.001 vs. OGD/R group; ^$^*p* < 0.05, ^$$^*p* < 0.01, ^$$$^*p* < 0.001 vs.OGD/R+BMSC-Exos group.

While TEM indicated the presence of a few autophagosomes in the control group, there were more autophagosomes in the OGD/R group. In contrast, a larger number of autolysosomes were detected in the BMSC-Exos group (Figures 4A,B).

Compared with the control group, the numbers of yellow puncta (GFP+RFP+) and red puncta (GFP-RFP+) increased in the OGD/R group. The numbers of red puncta further increased with BMSC-Exos treatment when compared with the OGD/R group, suggesting that BMSC-Exos treatment may promote autophagic flux. Furthermore, the autophagy inhibitor Cq reversed the results of GFP-RFP-LC3 staining and CCK-8 and LDH assays (Figures 4C–F).

**Figure 5:**
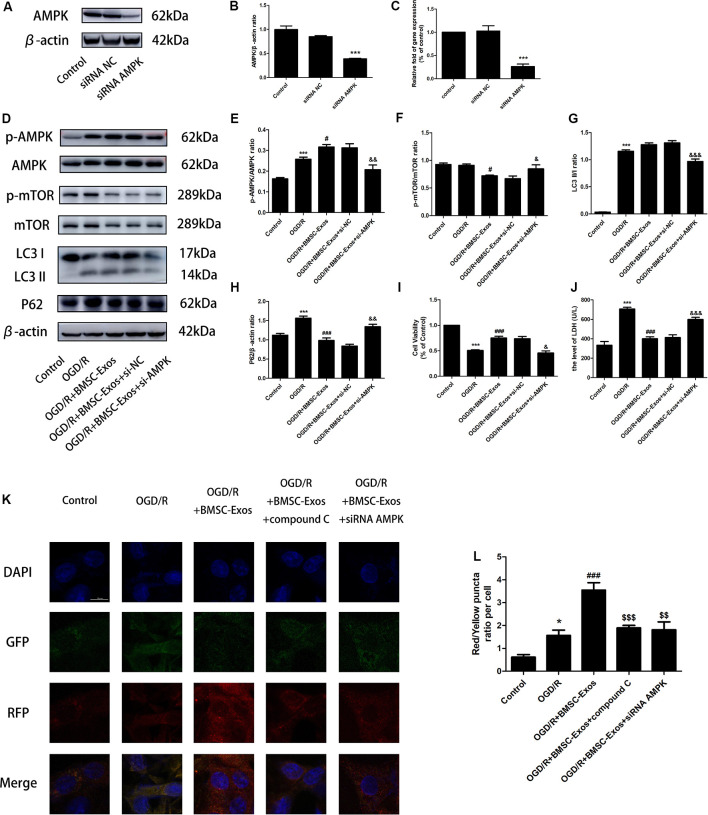
AMPK knockdown negated the effects of BMSC-Exos on autophagic flux. **(A–C)** AMPK protein levels were reduced following gene silencing. AMPK knockdown reversed the effects of BMSC-Exos on **(D–H)** activation of p-AMPK/AMPK and LC3 II/I, inhibition of p-TOR/mTOR and P62, **(I)** increase in cell viability, and **(J)** reduction in LDH release (*n* = 3). **(K,L)** AMPK knockdown reversed the effects of BMSC-Exos on the ratio of red puncta to yellow puncta (*n* = 3). *p < 0.05, ***p < 0.001 vs. control group; ^#^*p* < 0.05, ^###^*p* < 0.001 vs. OGD/R group; ^$$^*p* < 0.01, ^$$$^*p* < 0.001 vs. OGD/R+BMSC-Exos group; ^&^*p* < 0.05, ^&&^*p* < 0.01, ^&&&^*p* < 0.001 vs. OGD/R+BMSC-Exos+si-NC group.

The expression levels of LC3 II/I and p-AMPK/AMPK increased, whereas those of P62 and p-mTOR/mTOR decreased in the OGD/R group when compared with the control group. LC3 II/I expression did not change significantly after BMSC-Exos treatment, whereas p-AMPK/AMPK expression increased further and p-mTOR/mTOR and P62 expression levels decreased (Figures 4G–K). Moreover, AMPK knockdown by compound C and siRNA not only negated the effects of BMSC-Exos on AMPK, autophagic flux-associated proteins (Figures 4G–K, Figures 5A–H), and GFP-RFP-LC3 staining (5K,L) but also abrogated their ability to rescue PC12 cells from OGD/R-induced cell death and LDH release (Figures 4L,M, 5I,J).

### Blocked Autophagic Flux Reversed the Protective Effect of BMSC-Exos Against OGD/R-Induced Pyroptosis

**Figure 6:**
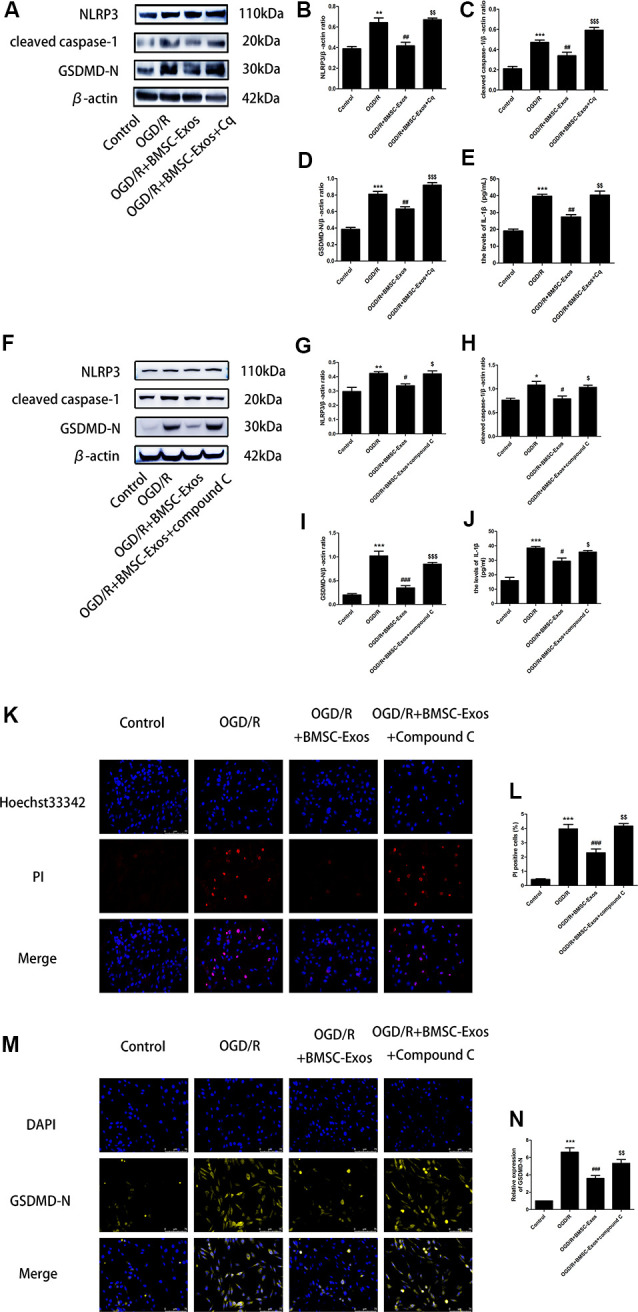
Blocked autophagic flux reverses the protective effect of BMSC-Exos against OGD/R-induced pyroptosis. **(A)** Representative Western blots of NLRP3, cleaved caspase-1, and GSDMD-N. **(B–D)** NLRP3, cleaved caspase-1, and GSDMD-N expression levels in the BMSC-Exos+Cq group were higher than in the BMSC-Exos group (*n* = 3). **(E)** IL-1β expression was higher in the BMSC-Exos+Cq group than in the BMSC-Exos group (*n* = 3). **(F)** Representative Western blots of NLRP3, cleaved caspase-1, and GSDMD-N. **(G–I)** NLRP3, cleaved caspase-1, and GSDMD-N expression levels in the BMSC-Exos+compound C group were higher than in the BMSC-Exos group (*n* = 3). **(J)** IL-1β expression was higher in the BMSC-Exos+compound C group than in the BMSC-Exos group (*n* = 3). **(K)** Representative images of Hoechst 33342/PI staining; scale bar = 75 μm. **(L)** Proportions of PI stained areas were higher in the BMSC-Exos+compound C group than in the BMSC-Exos group (*n* = 3). **(M)** Representative immunofluorescence images; scale bar = 75 μm. **(N)** Relative expression levels of GSDMD-N were higher in the BMSC-Exos+compound C group than in the BMSC-Exos group (*n* = 3). *p < 0.05, **p < 0.01, ***p < 0.001 vs. control group; ^#^*p* < 0.05, ^##^*p* < 0.01, ^###^*p* < 0.001 vs. OGD/R group; ^$^*p* < 0.05, ^$$^*p* < 0.01, ^$$$^ *p* < 0.001 vs. OGD/R+BMSC-Exos group.

**Figure 7:**
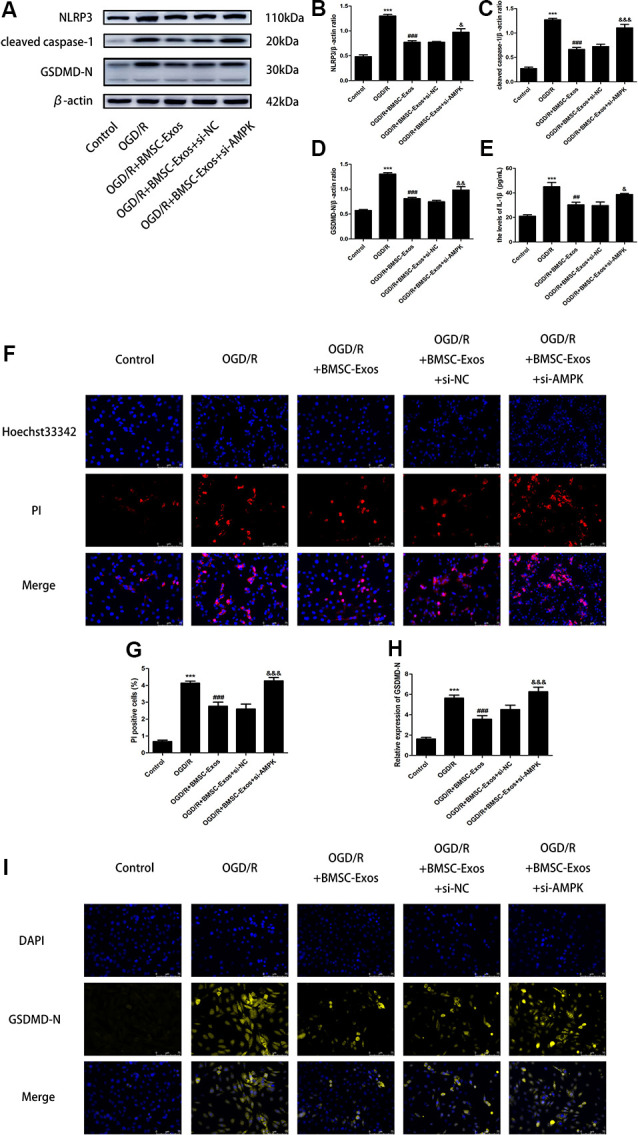
AMPK knockdown negated the protective effect of BMSC-Exos against pyroptosis. **(A–E)** AMPK knockdown reversed the effect of BMSC-Exos on inhibition of NLRP3, cleaved caspase-1, GDDMD-N, and IL-1β (*n* = 3). **(F,G)** AMPK knockdown reversed the effects of BMSC-Exos on the proportions of PI stained areas (*n* = 3). **(H,I)** AMPK knockdown reversed the effects of BMSC-Exos on the relative expression levels of GSDMD-N (*n* = 3). ***p < 0.001 vs. control group; ^##^*p* < 0.01, ^###^*p* < 0.001 vs. OGD/R group; ^&^*p* < 0.05, ^&&^*p* < 0.01; ^&&&^ *p* < 0.001 vs. OGD/R+BMSC-Exos+si-NC group.

To identify whether AMPK-dependent autophagic flux was involved in the effect of BMSC-Exos on the inhibition of cell pyroptosis, PC12 cells were pretreated with Cq, compound C, or transfected with AMPK-specific siRNA. Our results showed that Cq, compound C, and AMPK siRNA increased the expression of NLRP3, cleaved caspase-1, GSDMD-N, and IL-1β and reversed the results of the Hoechst 33342/PI double staining when compared with the OGD/R+BMSC-Exos group (Figures 6, 7).

**Figure 8:**
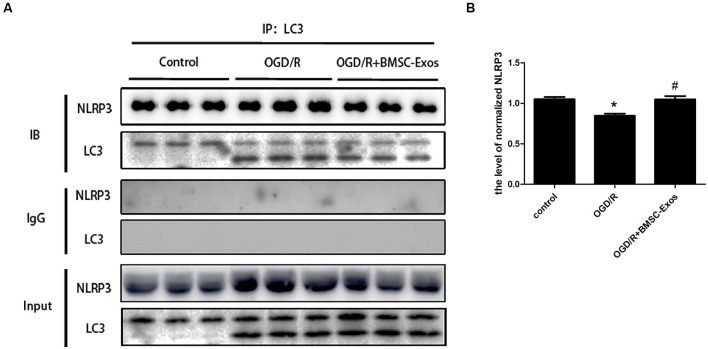
Coimmunoprecipitation of NLRP3 and LC3. **(A)** Cell lysates from PC12 cells under normal condition or OGD/R with or without BMSC-Exos were incubated with anti-LC3B antibody. NLRP3 and LC3 in precipitates were detected by Western blot. IgG was used as a negative control. **(B)** The NLRP3 was precipitated by anti-LC3B antibody, and the relative amount of NLRP3 was compared (*n* = 3 biological replicates per group). The direct interaction between NLRP3 and LC3 was significantly decreased under OGD/R, and BMSC-Exos could increase the interaction. *p < 0.05 vs. control group, ^#^*p* < 0.05 vs. OGD/R group.

We next detected the specific binding interaction between NLRP3 and LC3 in Co-IP assays. As shown in Figure 8, there was a direct interaction between NLRP3 and LC3. The direct interaction was significantly decreased under OGD/R, and BMSC-Exos could increase it.

## Discussion and Conclusion

**Figure 9:**
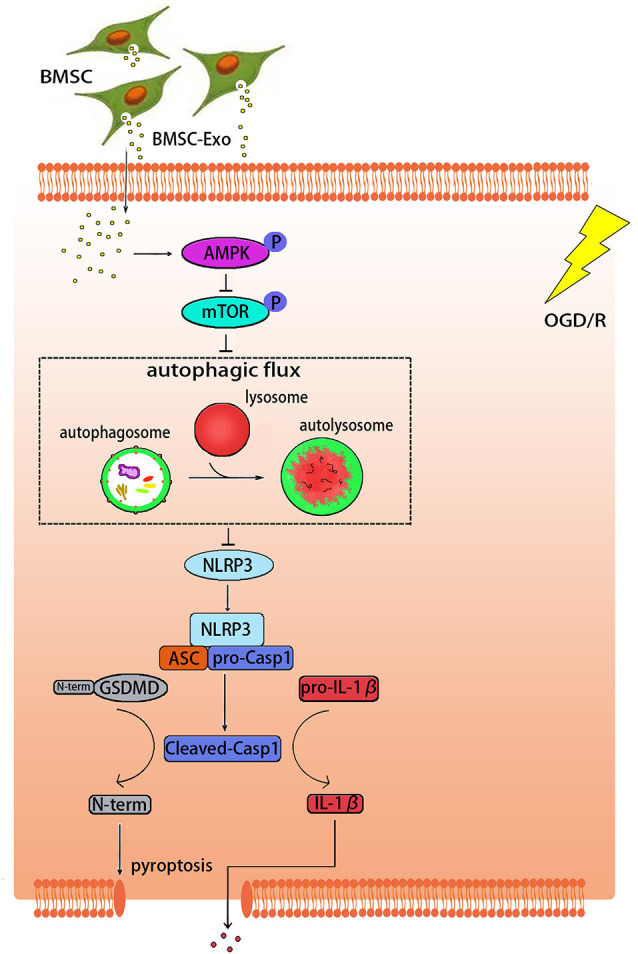
Graphical abstract demonstrating how BMSC-Exos inhibit NLRP3 inflammasome-mediated pyroptosis by promoting AMPK-dependent autophagic flux in OGD/R.

The pathological mechanism of cerebral I/R injury is complex and not well understood (Kalogeris et al., 2016). Among all the pathological factors, NLRP3 inflammasome-mediated pyroptosis has recently been considered as an important process in cerebral I/R injury (Zhou et al., 2019). In this study, we demonstrated that the protective effect of BMSC-Exos on OGD/R injury is related to the inhibition of NLRP3 inflammasome-mediated pyroptosis by promoting AMPK-dependent autophagic flux (Figure 9).

As an important component of paracrine signaling in stem cells, exosomes appear to be promising candidates for repairing tissue injury in place of stem cells (Lazar et al., 2018; Williams et al., 2020). Previous studies have found that BMSC-Exos can promote nerve function and improve the nervous system in ischemic stroke (Deng et al., 2019; Xiao et al., 2019; Hou et al., 2020; Safakheil and Safakheil, 2020), but the specific mechanism remains to be explored. In the current study, TEM, NanoSight NTA, and the surface marker proteins confirmed that the BMSC-Exos were successfully isolated and met international standards (Théry et al., 2018). CCK-8 and LDH assays further showed that BMSC-Exos at a dose of 10 μg/ml improved the viability of cells subjected to OGD/R, indicating that BMSC-Exos had a protective effect.

Pyroptosis, also known as inflammatory necrosis, is a new type of pro-inflammatory programmed cell death that plays an important role in cerebral I/R injury (Xia et al., 2018; Zhou et al., 2019; Zhu et al., 2019). This mode of cell death is mediated by caspase-1 and GSDMD, which induces the formation of pores in the cell membranes, resulting in the release of a large number of inflammatory cytokines (Coll et al., 2011; Lacey et al., 2018). Recent study has indicated that BMSC-Exos could protect against ischemic stroke through anti-inflammation and anti-apoptosis effects (Hou et al., 2020; Safakheil and Safakheil, 2020). In this study, we found that the protective effect of BMSC-Exos was associated with a reduced pyroptosis. Western blot and ELISA results further showed that BMSC-Exos inhibited the high expression of the key proteins associated with pyroptosis such as cleaved caspase-1, GSDMD-N, and IL-1β induced by OGD/R. Moreover, the PI staining proportion was decreased in the BMSC-Exos treatment group and LDH release was reduced significantly, indicating that the cell membranes of the cells that received the BMSC-Exos intervention were more complete than those of the OGD/R group. Importantly, SEM indicated that the cells treated with BMSC-Exos had clearer outlines, more dense surface villi, and longer protrusions, which were in contrast to the characteristics of pyroptosis in the OGD/R group.

As a component of pyroptosis, NLRP3 inflammasome is of concern because it can be activated by various PAMPs and DAMPs and it is closely related to various human diseases (Gong et al., 2018; Liu et al., 2018; Eren and Özõren, 2019). When NLRP3 is assembled and activated, caspase-1 can be activated and turn pro-IL-1β and GSDMD into the bioactive cytokines IL-1β and GSDMD-N, respectively (Hou et al., 2018). In this study, we found that the use of BMSC-Exos inhibited the high expression of NLRP3 and other key proteins associated with pyroptosis in OGD/R injury. This effect was similar to the NLRP3 inhibitor MCC950 and was reversed by NLRP3 overexpression. Various studies have found that ROS represents a key signal that regulates the activation of the NLRP3 inflammasome (Tschopp and Schroder, 2010; Eren and Özõren, 2019). We found that the ROS level in the cells subjected to OGD/R was increased significantly and that BMSC-Exos could weaken this response, indicating that BMSC-Exos reduced pyroptosis mediated by the NLRP3 inflammasome.

Autophagy is a dynamic process of degradation (Cardinal et al., 2009). The protective effect of BMSC-Exos on myocardial I/R injury appears to be closely related to the activation of autophagy (Zou et al., 2019), but their mechanism of autophagy in cerebral I/R injury has never been reported. In the current study, both the results of TEM and GFP-RFP-LC3 indicated that there was a large increase in number of autolysosomes in the OGD/R+BMSC-Exos group, further suggesting that BMSC-Exos was involved in the promotion of autophagic flux. In addition, the autophagy inhibitor Cq was shown to reverse the effect of activation and protection, indicating that the role of BMSC-Exos in reducing OGD/R injury was related primarily to their promotion of autophagic flux.

AMPK is a heterologous silk/threonine kinase distributed in many tissues where it regulates cellular energy, whose phosphorylation can also activate inhibitors of mTOR, thus activating autophagy (Qi and Young, 2015). Therefore, the AMPK/mTOR signaling pathway is an important regulatory pathway for autophagy (Puissant and Auberger, 2010; Jiang et al., 2018). In this study, BMSC-Exos treatment was found to activate AMPK phosphorylation and suppress mTOR phosphorylation during OGD/R. Both compound C and AMPK silencing reversed the promoting effect of BMSC-Exos on autophagic flux, which suggested that autophagic flux promoted by BMSC-Exos was AMPK-dependent.

Some recent studies have revealed that the protective effect of AMPK-dependent autophagy is partially related to the inhibition of pyroptosis. Li et al. (2019) found that adrenomedullin may protect the steroidogenic functions of Leydig cells against pyroptosis by activating autophagy *via* the ROS/AMPK/mTOR axis. In addition, Yang et al. verified that metformin could inhibit the NLRP3 inflammasome by activating the AMPK/mTOR pathway in diabetic cardiomyopathy (Yang et al., 2019). Our results showed that Cq reversed the inhibitory effect of BMSC-Exos on pyroptosis, suggesting that BMSC-Exos may alleviate cell pyroptosis by promoting autophagic flux. Moreover, both compound C and AMPK silencing reversed the effect described above, which suggested that autophagic flux promoted by BMSC-Exos for alleviating cell pyroptosis was AMPK-dependent. Co-IP experiments further showed a direct interaction between NLRP3 and LC3. The interaction was significantly decreased under OGD/R, and BMSC-Exos could increase it, which confirmed the impact of BMSC-Exos on the interaction between NLRP3 inflammasome and autophagy.

This study demonstrated that BMSC-Exos can protect PC12 cells against OGD/R injury through the attenuation of NLRP3 inflammasome-mediated pyroptosis by promoting AMPK-dependent autophagic flux. Further *in vivo* studies are warranted to verify the effect of BMSC-Exos on cerebral I/R injury.

## Data Availability Statement

The raw data supporting the conclusions of this article will be made available by the authors, without undue reservation, to any qualified researcher.

## Ethics Statement

The animal study was reviewed and approved by the Ethics Committee of Zhujiang Hospital, Southern Medical University.

## Author Contributions

QZ, YZ, and GH planned and conducted all the experiments. QZ, YZ, DL, HH, XL, RZ, and MZ contributed to performing the experiments. QZ and YZ contributed to performing the data analysis and drafting the manuscript. XL and YW revised the manuscript. All authors approved the final manuscript.

## Conflict of Interest

The authors declare that the research was conducted in the absence of any commercial or financial relationships that could be construed as a potential conflict of interest.
